# Supplementary material for: Brachygnathia Inferior in Cloned Dogs Is Possibly Correlated with Variants of Wnt Signaling Pathway Initiators
Source: Int J Mol Sci. 2022 Jan 1;23(1):475. doi: 10.3390/ijms23010475 (PMC8745273; doi:10.3390/ijms23010475)
Supplement: Supplementary file 1 [file ijms-23-00475-s001.zip › Supplemantary Tables S1-S3.pdf]

Supplementary Table S1. Results of sequencing quality.

Name	Pairs	Reads	Length(bp)	Avg.Qual	GC	DUP
OD	445,068,246	890,136,492	89,903,785,692	35	40.0	95
NT 1	376,391,542	752,783,084	76,031,091,484	35	40.0	86
NT 2	444,198,561	888,397,122	89,728,109,322	35	40.0	95
NT 3	470,391,857	940,783,714	95,019,155,114	34	40.0	83
NT 4	434,429,759	868,859,518	87,754,811,318	35	39.9	84

Pairs, Count of read pair; Reads, Total read count; Length, Total lenghth; Avg.Qual., average of sequence quality ; GC, GC Content; DUP, Total Duplication Percentage.

Supplementary Table S2. Results of mapping sequence

Sample	Raw	Trimmed<br>(%)	Mapped<br>(%)	Dedup<br>(%)	Depth
OD	89,903,785,692	89,650,467,408<br>99.7%	88,527,192,302<br>98.5%	84,375,551,803<br>93.9%	35.0x
NT 1	76,031,091,484	75,816,074,876<br>99.7%	74,783,520,294<br>98.4%	72,045,769,760<br>94.8%	29.9x
NT 2	89,728,109,322	89,461,444,841<br>99.7%	88,228,245,826<br>98.3%	84,221,546,412<br>93.9%	34.9x
NT 3	95,019,155,114	94,736,075,577<br>99.7%	93,409,043,586<br>98.3%	88,251,673,559<br>92.9%	36.6x
NT4	87,754,811,318	87,502,934,928<br>99.7%	86,179,435,833<br>98.2%	82,122,254,171<br>93.6%	34.1x

Raw, Total length of reads; Trimmed, Total length of trimmed reads; Mapped, Total length of mapped reads; Dedup, Total length of de-duplicated reads

Supplementary Table S3. Results of variations in genomic region

Sample	UP	5-UTR	Exon	Intron	Splice site	Tran	3-UTR	Down	Inter
					Acc	Don	Reg		
OD	519,176	11,339	74,791	2,815,251	7,078	19	32,954	483,479	3,828,492
					600	365	6,113		
NT 1	510,178	10,582	72,823	2,783,065	6,967	20	32,544	476,376	3,774,444
					593	346	6,028		
NT 2	518,512	11,074	74,208	2,813,433	7,085	22	32,942	483,186	3,827,407
					610	361	6,114		
NT 3	522,747	11,229	74,316	2,826,747	7,099	20	33,127	486,699	3,831,383
					613	347	6,139		
NT4	515,495	10,684	73,666	2,807,648	7,059	21	32,881	481,453	3,816,402
					598	350	6,111		

UP, Upstream; Acc, Splice Site Acceptor; Don, Splice Site Donor; Reg, Splice Site Region; Tran, Transcript; Down, downstream; Inter, Intergenic
