# Supplementary material for: Brachygnathia Inferior in Cloned Dogs Is Possibly Correlated with Variants of Wnt Signaling Pathway Initiators
Source: Int J Mol Sci. 2022 Jan 1;23(1):475. doi: 10.3390/ijms23010475 (PMC8745273; doi:10.3390/ijms23010475)
Supplement: Supplementary file 1 [file ijms-23-00475-s001.zip › Supplementary Figure S1.pdf]

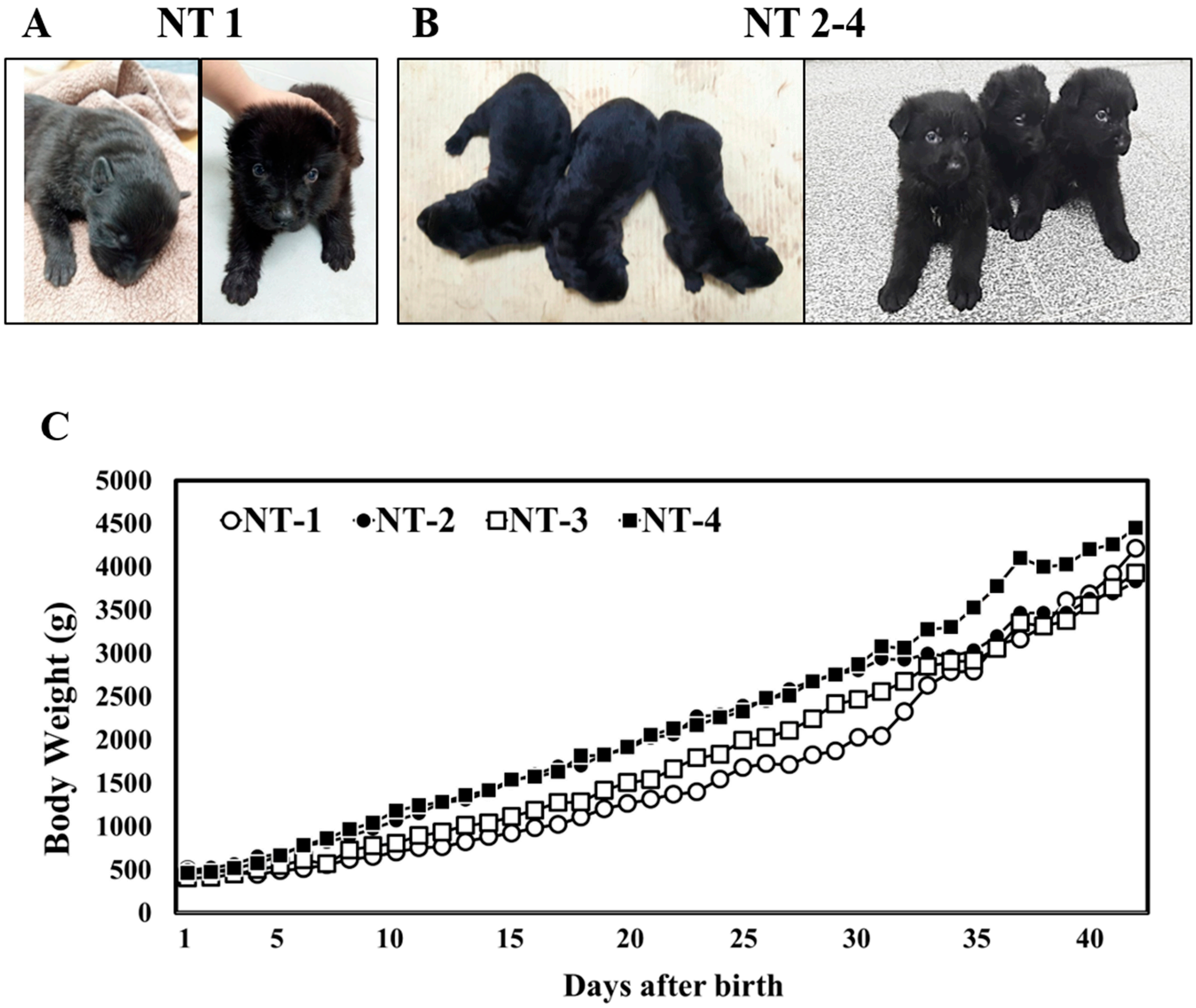

Supplementary Figure S1. Production of cloned newborn puppies and daily body weight monitoring of cloned puppies.

(**A**) NT-1 obtained by caesarian section of SMD1, (**B**) puppies (NT-2, 3 and 4 from SMD2) at the age of 1 day and ~4 weeks, (**C**) body weights of NT-1~4 from one day to 42 days after birth.
